# Comparison of the Efficacy of Duloxetine and Pregabalin in Pain Relief Associated with Diabetic Neuropathy

**DOI:** 10.7759/cureus.5293

**Published:** 2019-07-31

**Authors:** Wajeeha Shahid, Ravi Kumar, Anam Shaikh, Sham Kumar, Rakhshinda Jameel, Sundus Fareed

**Affiliations:** 1 Internal Medicine, Jinnah Sindh Medical University, Karachi, PAK; 2 Internal Medicine, Dow University of Health Sciences (DUHS), Karachi, PAK; 3 Internal Medicine, Civil Hospital Karachi, Karachi, PAK; 4 Internal Medicine, Australian Concept Infertility Medical Center, Karachi, PAK

**Keywords:** duloxetine, pregabalin, diabetic neuropathy, efficacy, tolerability, open label trial, painful diabetic peripheral neuropathy, vas score

## Abstract

Introduction

Painful diabetic peripheral neuropathy (PDPN) complicates 25% of type II diabetes mellitus. It has a profound impact on diabetes-related morbidity and worsens the quality of life. Both pregabalin and duloxetine may be indicated for PDPN. In this study, the efficacy of duloxetine and pregabalin was compared in patients with PDPN.

Methods

It was a single-centre open-label study conducted with patients of diabetes mellitus type II diagnosed with PDPN. Patients were randomized to receive 60 mg/daily duloxetine or 300 mg/daily pregabalin. Pain scores were recorded using visual analogue scale (VAS) on day 0, week 4, and week 12. Data was entered and analysed using SPSS version 22.0 (IBM Corp., Armonk, NY).

Results

In the duloxetine group, the mean VAS score decreased from 6.81 ± 0.91 to 4.01 ± 1.12 with 12 weeks of therapy (p <0.0001). In the pregabalin group, the mean VAS score decreased from 6.99 ± 1.12 to 4.91 ± 0.82 with 12 weeks of therapy (p <0.0001). At 12 weeks, duloxetine showed lower VAS scores than pregabalin (p <0.0001). In the duloxetine group, the mean change in VAS score over time was - 2.80 and in the pregabalin group, the mean change was - 2.80. Adverse events were reported in 17.9% of the participants. Lethargy/somnolence (8.1%) and peripheral edema (3.4%) were commonly reported in the pregabalin group and constipation (6.9%) and orthostatic hypotension (4.6%) were commonly reported in the duloxetine group.

Conclusions

Duloxetine at a daily fixed dose of 60 mg is efficacious in the relief of neuropathic pain. Pregabalin also showed a comparable outcome. Both duloxetine and pregabalin have a promising safety profile and are well-tolerated.

## Introduction

Chronic painful diabetic peripheral neuropathy (PDPN) is a common, debilitating, and distressing complication of type II diabetes mellitus [1]. Diabetic neuropathy may be triggered by neuronal apoptosis and inhibition of nerve regeneration which is due to chronic hyperglycemia and may lead to significant deficits in tactile sensitivity, vibration sense, lower-limb proprioception, and kinaesthesia [2]. PDPN occurs in as many as 25% of patients with diabetes mellitus [3]. The most common form of PDPN is the “distal sensorimotor polyneuropathy.” PDPN is predominantly characterized by sensory symptoms in the “glove-and-stocking” distribution [4]. In addition to directly causing pain, paresthesia, numbness, and insensitivity, it can also impair sleep, depress mood, increase the risk for foot injuries and burns, and hinder everyday activities, resulting in a poor quality of life [5-6].

Once the diagnosis of PDPN is established, one of the two therapeutic approaches may be ensued: pathogenic treatments targeting the underlying pathophysiological processes to prevent nerve fibre loss; and symptomatic treatments aiming to alleviate the painful symptoms which helps in improving physical and psychological functioning [7]. The symptomatic management of neuropathic pain in diabetes remains a major management challenge for clinicians and a number of clinical guidelines have evolved to guide clinicians as to the most effective treatments for these patients [7]. Both duloxetine and pregabalin are approved as the first-line for treatment of PDPN as per international guidelines [8-9].

Duloxetine is a serotonin and norepinephrine reuptake inhibitor. Its mechanism of action is related to the potentiation of serotonergic and noradrenergic activity in the descending inhibitory pain pathways of the central nervous system [10]. Pregabalin belongs to the class of anticonvulsants. It reduces the release of excitatory neurotransmitters involved in pain perception by binding to presynaptic neuronal calcium channels [11]. Both pregabalin and duloxetine have been recommended as the initial approach in the symptomatic treatment. Although both are readily being used for management of PDPN in Pakistan, to the best of our knowledge, there was no head-to-head comparison between the two drugs from Pakistan. This study aimed to compare the efficacy of duloxetine with pregabalin in patients with painful diabetic neuropathy in a tertiary care hospital in Pakistan.

## Materials and methods

It was an open-label prospective trial conducted in a public tertiary care hospital in Karachi. The study duration was from January to March 2019. The study was conducted in the outpatient diabetes clinic after approval from the Institutional Review Board. Informed consent was attained from all participants.

During the study period, all patients of diabetes mellitus type II, diagnosed with PDPN were included after informed consent. The diagnosis of PDPN is primarily clinical, based on a history of neuropathic pain and confirmatory examination findings, establishing deficits associated with neuropathy. All patients were diabetic, with history of pain and numbness in hands and feet in "gloves and stocking" pattern. Biothesiometry was done for all patients. Biothesiometer score of 16 volts or above was diagnostic for PDPN. Participants were randomized into two groups. Group A included patients who received duloxetine 60 mg/daily and Group B included patients who received pregabalin 300 mg/day. All patients were naïve to their respective drug. Patients with diabetes-related foot injuries, ulcers, and/or any other painful wound/lesion were excluded as they would’ve created a bias in pain scores. Patients with any other diabetes-related complication such as nephropathy, retinopathy and/or cardiopathy were also excluded. Patients who couldn’t understand and/or communicate in English were also excluded.

Neuropathic pain was evaluated using the visual analogue scale (VAS). Scores were based on self-reported measures of pain intensity ranging from 0: no pain to 10: worst pain. A higher score indicates greater pain intensity [12]. Patients were required to respond to VAS on three instance - day zero, week four, and week 12 of the study. Only complete responses with VAS input on all three instances were included in the study. The occurrence of adverse events was also recorded in all instances. Demographic characteristics of the patients including age and gender were recorded. Disease related characteristics including duration of diabetes and their treatment.

At the beginning of the study, 193 participants were invited. Twenty of these were not included either due to lack of consent or language barrier. The remaining 173 were randomized to Group A (n=87) and Group B (n=86). On week four, no patient was lost to follow up in group A and two were lost in group B. By week 12 follow up, two patients had stopped taking the medication in group B due to adverse events. Another three patients were lost to follow up in group B by week 12 follow up and five in group A. Hence, 12-week study was completed by 161 patients - 82 in group A and 79 in group B. Data was processed through and analysed using SPSS for Windows, version 22.0 (IBM Corp., Armonk, NY). Mean and standard deviation (SD) was calculated for continuous variables and frequencies and percentages were calculated for categorical variables. For analysis of mean scores of VAS only patients who completed the study (n=161) were included, and for adverse events, all 173 patients were included.

## Results

Among the 161 patients, there were 93 (%) men and 68 (%) women. Their mean age was 63 ± 7 years (range: 54-70 years). The mean duration of diabetes was 9.4 ± 3.5 years (range: 6-14 years). The duloxetine group had a longer mean duration of diabetes than the pregabalin group (10.3 ± 1.4 vs. 7.5 ± 1.9 years).

Mean pain intensity recorded on VAS for duloxetine and pregabalin group on day 0, week 4, and week 12 are shown in Table 1. There was a statistically significant reduction in pain over time within the groups. At week 12, the duloxetine group showed significantly lower VAS score as compared to pregabalin group. Mean change in pain score is also shown which indicates a higher decrease in pain intensity in the duloxetine group; however, the difference was not significant between the groups (p=0.90) (Table 1).

**Table 1:** Mean scores of pain intensity on day 0, week 4, and week 12 in the duloxetine and pregabalin groups (N=161) *Means of day 0 and week 12 within the group were compared; **Means of week 12 for both groups were compared SD: Standard deviation; VAS: Visual analog scale.

	Day 0	Week 4	Week 12	P value		Mean Change	
VAS Score (Mean ± SD)				Intra group*	Inter group**	Intra group*	Inter group**
Duloxetine (n=82)	6.81 ± 0.91	5.60 ± 0.89	4.01 ± 1.12	<0.0001	<0.0001	– 2.80	0.90
Pregabalin (n=79)	6.99 ± 1.12	6.01 ± 0.82	4.91 ± 0.82	<0.0001		– 2.08	

Adverse events were reported in 31/173 (17.9%) participants. Two participants of the pregalabin group discontinued treatment due to adverse events. Both were males and both complained of sexual dysfunction. No patient in the duloxetine group discontinued therapy. The most common adverse events reported in the pregabalin group were lethargy/somnolence, peripheral edema, and constipation. The commonly reported adverse events in the duloxetine group were orthostatic hypotension and constipation. The frequency of adverse events reported in both groups is shown in Table 2.

**Table 2:** Frequency of adverse events reported in both study groups (N=173)

Adverse Event	Duloxetine (n=87)	Pregabalin (n=86)
Discontinuation due to adverse event	-----	2 (2.3%)
Lethargy / Somnolence	1 (1.1%)	7 (8.1%)
Decrease appetite	2 (2.2%)	2 (2.3%)
Peripheral edema	-----	3 (3.4%)
Vomiting	3 (3.4%)	-----
Constipation	6 (6.9%)	3 (3.4%)
Sexual dysfunction	-----	2 (2.3%)
Blurred Vision	1 (1.1%)	-----
Orthostatic Hypotension	4 (4.6%)	-----

## Discussion

Both duloxetine and pregabalin have shown a significant reduction in neuropathic pain over a period of 12 weeks. Duloxetine showed a higher reduction in pain as compared to pregabalin and the differences were statistically significant.

While this study, to the best of our knowledge, is the first local study to compare pregabalin and duloxetine for pain reduction in diabetic peripheral neuropathy, it has its limitations. First, since it was an open-label trial, it may have introduced bias from both the patients as well as the doctors. The results cannot be projected for a longer duration as it was only a 12-week-long study. Furthermore, being a single centre study, its results cannot be extrapolated for the general population. This study has rolled out preliminary results and generates the need to conduct a large scale study with a robust methodology.

In this study, duloxetine showed better pain reduction as compared to pregabalin. From day 0 to week 12, duloxetine showed -2.8 point mean reduction in VAS score. This result was comparable to Tanenberg et al. [13] where patients treated with duloxetine had significantly greater pain reduction than pregabalin at week 4 and at each successive week up to the 12-week endpoint. Goldstein et al. in their work also established the efficacy and safety of duloxetine in PDPN. Duloxetine 20 mg/day failed to produce any significant pain reduction as compared to placebo, however, duloxetine 60 mg and 120 mg per day did so. Furthermore, 10.7% of patients discontinued duloxetine due to adverse events [14]. None of the patients in the duloxetine group did so in this study. In a study by Raskin et al., 12.1% of patients discontinued duloxetine due to adverse events [15]. However, in another open-label safety trial, discontinuation because of adverse events was significantly greater in the duloxetine (19.6%) vs. pregabalin group (10.4%; p = 0.04) [16]. Lethargy, somnolence, edema, blurred vision were found more in pregabalin group which is consistent with findings of Freynhagen [17] which reported that most common adverse event associated with pregabalin are dizziness, peripheral odema, weight gain, and somnolence. Constipation and hypotension were more common in the duloxetine group which is consistent with findings of previous studies [14-15].

In a double-blind randomized trial, both the duloxetine and pregabalin treatment groups showed improvement in neuropathic pain from baseline; there were no significant differences between the two groups. Both drugs were well tolerated [18]. Similarly, in an open-label trial conducted in India, a significant reduction in pain score on VAS was seen in all the three treatment groups (duloxetine, pregabalin, and gabapentin) across time (p <0.05). Pain improvement was higher in the pregabalin group as compared to the duloxetine group; however, the differences were not statistically significant. They report adverse events in 9% of patients; none was serious enough to require discontinuation of therapy. In the duloxetine group, an increased urinary frequency was observed and in the pregabalin group, dizziness and nausea vomiting was reported [19].

Both duloxetine and pregabalin produce clinically meaningful pain relief. The efficacy of both drugs is comparable. Both the drugs are well tolerated. The choice of drug may be determined by clinical as well as patient factors.

## Conclusions

Duloxetine at a daily fixed dose of 60 mg is efficacious in the relief of neuropathic pain. Pregabalin also showed a comparable outcome. Both duloxetine and pregabalin have a promising safety profile and are well-tolerated. Multi-centre, longitudinal, double-blind trials must be ensued to establish robust evidence on the superiority of either drug in the management of PDPN.
